# Antigenic characterization of novel H1 influenza A viruses in swine

**DOI:** 10.1038/s41598-020-61315-5

**Published:** 2020-03-11

**Authors:** Rodrigo Tapia, Montserrat Torremorell, Marie Culhane, Rafael A. Medina, Víctor Neira

**Affiliations:** 1 Departamento de Medicina Preventiva Animal, Facultad de Ciencias Veterinarias y Pecuarias, Universidad de Chile, Santiago, 8820808 Chile; 2 Department of Veterinary Population Medicine, College of Veterinary Medicine, University of Minnesota, St. Paul, 55108 USA; 3 Departamento de Enfermedades Infecciosas e Inmunología Pediátrica, Escuela de Medicina, Pontificia Universidad Católica de Chile, Santiago, 8330024 Chile; 4 Department of Microbiology, Icahn School of Medicine at Mount Sinai, New York, 10029 USA

**Keywords:** Influenza virus, Viral immune evasion

## Abstract

Novel H1N2 influenza A viruses (IAVs) in swine have been identified in Chile co-circulating with pandemic H1N1 2009-like (A(H1N1)pdm09-like) viruses. The objective of this study was to characterize antigenically the swine H1 IAVs circulating in Chile. Genetic analysis based on the HA1 domain and antigenic analysis by hemagglutination inhibition assay were carried out. Three antigenic clusters were identified, named Chilean H1 A (ChH1A), Chilean H1 B (ChH1B), and A(H1N1)pdm09-like. The antigenic sites of ChH1A and ChH1B strains were 10–60% distant from those of commercial vaccine strains at the amino acid sequence level. Antigenic variants were identified within the clusters ChH1A and A(H1N1)pdm09-like. Substitutions in the main antigenic sites (E153G in Sa, Q193H in Sb, D168N in Ca1, P137S in Ca2, and F71L in Cb) were detected in variants from the ChH1A cluster, whereas only a single substitution in antigenic site Sa (G155E) was detected in variants from A(H1N1)pdm09-like cluster, which confirms the importance to carrying out antigenic analyses in addition to genetic analyses to evaluate control measures such as vaccination. These results highlight the need to update vaccines for swine in Chile and the importance of continued surveillance to determine the onward transmission of antigenic variants in Chilean pig populations.

## Introduction

Influenza A virus (IAV) is a member of the family *Orthomyxoviridae* possessing 8 negative sense single-stranded RNA segments^1^ and classified in subtypes based on the antigenicity of their surface glycoproteins: 18 subtypes for hemagglutinin (HA) and 11 subtypes for neuraminidase (NA)^2^. IAV can infect birds and several mammalian species, including human and swine. Pigs have an important role in the ecology of IAV, since they can become infected with both human and avian strains^3,4^. Co-infection with IAVs from different lineages can generate reassortant strains with potential epidemic and zoonotic risks^5–7^.

IAVs are ubiquitous in swine worldwide, generating significant economic losses and representing a public health concern^8,9^. The main control measure in many swine farms is the use of vaccines, but the commercial vaccines currently available are based on North American or European IAV strains^10,11^. H1N1, H1N2 and H3N2 are the main subtypes circulating in swine globally; however, IAVs in swine are genetically and antigenically diverse even within each subtype, and several lineages have been reported^12,13^. This IAV diversity is the result of genetic evolution and antigenic changes that occur primarily through 2 mechanisms: ‘antigenic shift’, by reassortment of gene segments encoding surface glycoproteins, HA and NA; and ‘antigenic drift’, by non-synonymous substitutions in these glycoproteins, mainly in the antigenic sites of the HA, against which neutralizing antibodies against IAV are predominantly generated^14,15^.

The HA is organized as a non-covalent homo-trimer on the viral surface where each monomer consists of two polypeptides, HA1 and HA2^16^. HA1 is the major immunogenic polypeptide, containing the receptor binding site (RBS) surrounded by five antigenic sites: Sa, Sb, Ca1, Ca2 and Cb, described for subtype H1^17,18^. Accumulation of amino acid substitutions in these antigenic sites (antigenic drift), due to immune selection pressure, can allow the virus to escape the preexisting immunity (antigenic variants or escape mutants), providing it a fitness advantage and the opportunity to emerge as a novel epidemic strain^15,19^. Moreover, some amino acid substitutions can result in the acquisition of glycosylation sites in the HA, some of which are maintained, while others are replaced or disappear over time^20^. Glycosylations in HA1 are known to be able to modulate the antigenicity, fusion activity, virulence, receptor-binding specificity, and immune evasion of IAV^21^. The immune evasion occurs when glycosylations appear in or near antigenic sites, interfering with the recognition of the virus by neutralizing antibodies^22,23^.

In Chile, despite limited information about IAV circulation in swine, North American commercial vaccines have been widely used in swine farms in the last years. However, only the pandemic H1N1 2009-like (A(H1N1)pdm09-like) IAVs, with H1s of global clade 1A.3.3.2^13^, are present in both Chile and in North America. There are recent studies that identified novel reassortant H1N2 IAVs of swine origin in Chile with H1s of global clade “Other-Human-1B.2” that are genetically distinct from the clade 1B.2 strains of IAV in North American pigs. These novel H1N2 viruses have A(H1N1)pdm09-like virus internal genes associated with HA and NA genes that are genetically divergent from all other IAVs identified in swine and humans globally. These novel HA and NA lineages are most related to human seasonal H1N1 viruses from the late 1980s and early 1990s, and they were likely introduced from human into swine during those decades^24–26^.

Although some IAV strains from pigs in Chile have been genetically characterized, the antigenic diversity of IAVs circulating in Chilean swine populations is unknown. The antigenic characterization is essential to evaluate the efficacy of diagnostic tests and current vaccines, to select suitable vaccine strains for the efficient control of disease in swine, and to assess the risk of IAV introductions from swine into the human population. Hence, we characterized antigenically the H1 IAVs circulating in swine farms between 2013 and 2015. We also analyzed the genetic basis for the antigenic differences among circulating H1 IAVs, identifying glycosylations and amino acid substitutions in or near antigenic sites of HA that may lead to immune evasion.

## Methods

### Viruses

Thirty-eight H1 IAV isolates (17 H1N2 and 21 H1N1) and their HA nucleotide sequences were obtained from 1,680 samples (1,411 nasal swabs, 263 oral fluids, and 6 lung tissues) collected in 36 swine farms, belonging to 22 companies, which represent >90% of the pig inventory in Chile (Supplementary Table S1). The farms were integrated one-site or multi-site farming systems with greater than 800 sows in the farrowing units. Samples were collected between December 2013 and December 2015, during a passive and active surveillance program carried out by Universidad de Chile and Pork Producers Trade Association of Chile (ASPROCER).

Nasal swab and oral fluid samples were obtained from pigs of 6 to 14 weeks, the age at which it is most likely to detect IAV-positive pigs^27^. Nasal swabs (FLOQSwabs™, Copan Diagnostics Inc., Murrieta, CA, USA) were inserted into both nostrils and placed in sterile tubes with 2 mL of IAV growth medium (MEM 1x supplemented with 1 μg/mL of trypsin treated with N-tosyl-L-phenylalanyl chloromethyl ketone (TPCK), 0.3% bovine serum albumin, and 1% antibiotic-antimycotic solution). Oral fluids were collected using 1 m of twisted cotton rope hanging for 30 min in each pen. Ropes were then squeezed into a sterile plastic bag and the oral fluids were deposited into a 50-mL conical centrifuge tube. Furthermore, farms enrolled in this study also submitted samples for diagnostic workups, including lung tissue, when suspected cases of respiratory disease due to IAV were observed.

All samples were centrifuged for 8 min at 6,000 × g and 4 °C (lung tissue samples were previously macerated in IAV growth medium) and the supernatants were used for virus isolation. Two hundred microliters of each sample were absorbed onto Madin-Darby Canine Kidney (MDCK) cells for 1 h at 37 °C and 5% CO_2_. Cells were then rinsed with 1x phosphate-buffered saline (PBS) to remove unbound virus, and IAV growth medium was added. Cells were incubated at 37 °C and observed for cytopathic effect (CPE) daily for 5 days^26^. CPE positive samples were tested by real time RT-PCR to confirm the presence of IAV. RNA was extracted with TRIzol® LS Reagent (Invitrogen™, Carlsbad, CA, USA) following the manufacturer indication, and a real time RT-PCR based on highly conserved regions of the IAV matrix gene was then carried out^28^.

HA gene sequences of a selection of IAV isolates per farm were obtained. The sequencing was performed at the Veterinary Diagnostic Laboratory of the University of Minnesota (Saint Paul, MN, USA) and at the Sequencing Core Laboratory of the Center for Research on Influenza Pathogenesis (CRIP), Icahn School of Medicine at Mount Sinai (New York City, NY, USA). The GenBank accession numbers for these sequences are provided in Supplementary Table S2.

### Genetic analysis

We analyzed nucleotide and amino acid sequences (deduced from nucleotide sequences) encoding the HA1 domain (327 amino acids), where the major antigenic sites of HA are located (Sa, Sb, Ca1, Ca2, and Cb for subtype H1)^18^. We used the N-terminal sequence of a mature protein (i.e. signal peptide sequence was not considered in the analysis) according to the recommended numbering scheme for influenza A HA subtypes^29^. The sequence alignment was done with MUSCLE using the MEGA software (version 7.0.26). The phylogenetic tree was constructed with Maximum likelihood method and General Time Reversible model with a variation rate among sites given by gamma distribution with invariant sites (GTR + G + I) using RaxML-HPC2 (version 8.2.12)^30^. Bootstrap values were determined with 1,000 replicates. Then, we computed pairwise distances between amino acid sequences only from the antigenic sites or the entire HA1 region, using the method p-distance in MEGA. A 3-dimensional (3D) genetic map was made with Multidimensional Scaling (MDS) method from a dissimilarity matrix based on HA1 amino acid sequences, and the genetic clusters in the map were defined by Ward’s method based on the Euclidean distances among strains, using the XLSTAT software (version 2018.1). Potential N-glycosylation sites were predicted on the HA1 domain by the motif N-X-S/T, using the NetNGlyc 1.0 Server (http://www.cbs.dtu.dk/services/NetNGlyc/). Chilean swine IAV sequences, North American swine IAV sequences (from H1 clusters used in commercially available vaccines: α (clade 1A.1), β (clade 1A.2), γ (clades 1A.3.2 and 1A.3.3.3), δ (clades 1B.2.1, 1B.2.2, 1B.2.2.1 and 1B.2.2.2) and pandemic (clade 1A.3.3.2)), and worldwide human IAV sequences were included in the analyses. Reference sequences were obtained from GenBank (https://www.ncbi.nlm.nih.gov/genbank/), BLAST (https://blast.ncbi.nlm.nih.gov/Blast.cgi), Influenza Research Database (https://www.fludb.org), and GISAID (https://www.gisaid.org/).

Finally, within the genetic clusters we selected 13 representative strains to produce antisera. To select these strains, we obtained the consensus amino acid sequence of the antigenic sites within each genetic cluster, using the Jalview software (version 2.10.0). Then, distance matrices (p-distance) were performed between the amino acid sequences of each genetic cluster and their respective consensus sequence using MEGA. We selected the strains with the highest amino acid identity to the consensus sequence of each genetic cluster, representing different farms, companies, and geographical regions.

### Antiserum production

Each selected virus containing at least 128 hemagglutination units (HAU)/50 μL, determined by a standard hemagglutination assay^31^, was inactivated with 0.1% formalin and supplemented (1:1) with complete Freund’s adjuvant for the first vaccination, and incomplete Freund’s adjuvant for the second vaccination.

We used guinea pigs to produce the antisera, because there is no source of influenza-negative pigs in Chile. The guinea pig has been commonly used as a model for immunization studies against IAVs^32–34^. Four-week-old female guinea pigs, Pirbright strain, free of IAV and IAV antibodies, were obtained from the Instituto de Salud Pública de Chile. They were acclimated for 7 days on a 12 h light/dark cycle and were allowed access to food and water *ad libitum*^26^. Animals were then randomly separated in experimental groups of 2 animals, which were immunized subcutaneously with 0.2 mL of each inactivated IAV vaccine. A second dose was applied 14 days after. Animals in a control group were injected with sterile PBS and adjuvant. Blood samples were collected by cardiac puncture 28 days post first vaccination after anesthesia with a mixture of ketamine (30 mg/kg) and xylazine (2 mg/kg) administered intramuscularly^35^. Animals were euthanized immediately after the blood sample collection with thiopental sodium (120 mg/kg) administered intraperitoneally. Blood samples were centrifuged for 10 min at 800 × g and 4 °C, and sera were collected and stored at −20 °C. All animal procedures were conducted in biosafety level 2 conditions and were approved by the Institutional Animal Care and Use Committee of Universidad de Chile, under protocol number 02-2016.

### Antigenic characterization

Hemagglutination inhibition (HI) assays were performed by testing the antisera against the 38 IAV strains, following a standard protocol^31^. Prior to HI assay, sera were treated to remove natural nonspecific hemagglutination inhibitors following a previously described protocol^36^. Briefly, 100 µL of each serum was heat inactivated at 56 °C for 30 min and 600 µL of 25% kaolin in borate saline was added. This mixture was incubated at room temperature for 30 min, and 600 µL of 25% turkey red blood cells were added. After 2 h of incubation at room temperature, the mixture was centrifuged, and the antisera were collected and stored at −20 °C until usage. All viruses were titrated using hemagglutination assay to quantitate their concentration and were diluted to 8 HAU/50 μL. A preliminary HI assay was performed with each antiserum and the corresponding homologous virus, in order to dilute and standardize each antiserum at HI titer of 320–640. The HI results were analyzed using the antigenic cartography software (http://www.antigenic-cartography.org/) to construct a 3D antigenic map. HI titer ≤20 was considered negative. Antigenic clusters were defined by Ward’s method based on the Euclidean distances among strains in the antigenic map, using the XLSTAT software.

### Structure of IAV HA

PyMOL Molecular Graphics System (version 2.1.1) was used to visualize and identify glycosylation sites and key amino acid substitution positions in the HA1 domain of Chilean swine H1 IAV strains. Genetically closest HA sequences to Chilean H1 clusters available in the RCSB PDB Protein Data Bank (https://www.rcsb.org/) were used. Full-length HA 3D structure of A/Thailand/CU44/2006(H1N1) (PDB ID: 4edb) was used for Chilean H1 A and Chilean H1 B clusters, while the HA 3D structure of A/California/04/2009(H1N1) (PDB ID: 3lzg) was used for the A(H1N1)pdm09-like cluster.

## Results

### Chilean H1 swine IAVs antigenic sites are genetically distant from vaccine strains

**Figure 1:**
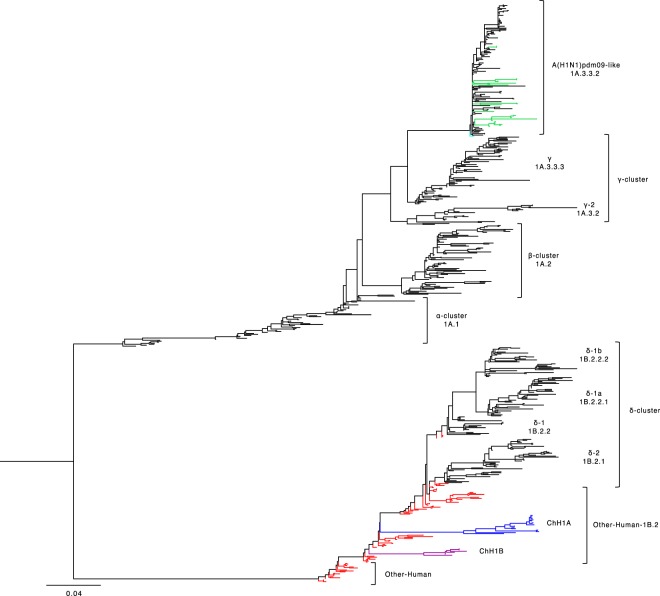
Chilean H1 swine IAVs are genetically distant from North American commercial vaccine strains. Phylogenetic tree based on HA1 domain nucleotide sequences of H1 swine IAVs, obtained using the Maximum Likelihood method and General Time Reversible model with a variation rate among sites given by gamma distribution with invariant sites (GTR + G + I). Five hundred eighty-nine sequences were used to construct this phylogenetic tree. Chilean swine IAVs (including reference sequences) are grouped in three phylogenetic clusters: Chilean H1A (ChH1A; blue), Chilean H1B (ChH1B; purple) and pandemic H1N1 2009-like (A(H1N1)pdm09-like; green). Reference sequences from human seasonal IAVs (1977–2008) are in red, and North American H1 swine clusters (α clade 1A.1; β clade 1A.2; γ clades 1A.3.2 and 1A.3.3.3; δ clades 1B.2.1, 1B.2.2, 1B.2.2.1 and 1B.2.2.2; and A(H1N1)pdm09-like clade 1A.3.3.2), used in commercial vaccines, are in black. The pandemic vaccine strain A/California/04/2009(H1N1) is highlighted in cyan.

**Figure 2:**
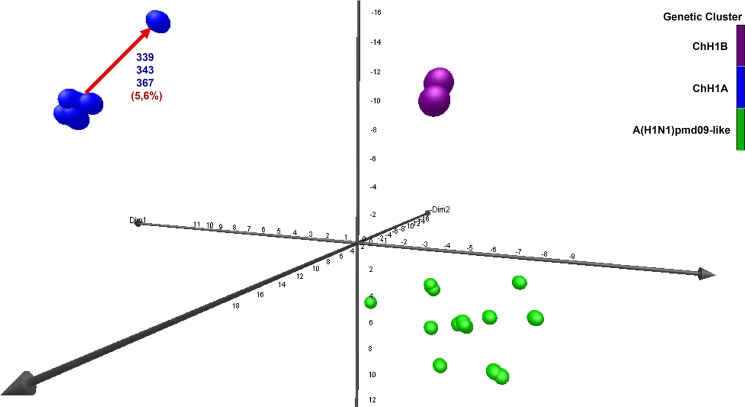
Genetic analysis of Chilean H1 swine IAVs based on HA1 amino acid sequences. A three-dimensional genetic map was constructed with HA1 domain amino acid sequences. All axes represent amino acid distance (percent of distance), and the orientation of the map within these axes is free. Circles represent the 38 swine IAV strains used in this study and genetic clusters in the map were defined by Ward’s method based on the Euclidean distances among these strains. Color represents the genetic clusters: ChH1A is blue, ChH1B is purple, and A(H1N1)pdm09-like is green. Value in percentage (%) and red arrow shows the distance between the center of the ChH1A cluster and their antigenic variants A/swine/Chile/VN1401-339/2014(H1N2) (339), A/swine/Chile/VN1401-343/2014(H1N2) (343) and A/swine/Chile/VN1401-367/2014(H1N2) (367).

We used the HA1 domain sequences from 38 swine IAV isolates to determine the Chilean genetic clusters and compared them with North American swine IAV clusters used to produce the current commercially available vaccines. Genetic representatives of vaccine strains were used, since the actual strain sequences are proprietary information, with the exception of the A(H1N1)pdm09 strain A/California/04/2009(H1N1) that is publicly available. The phylogenetic tree and the genetic map show that the Chilean H1 swine IAV sequences were grouped in 3 clusters, named: Chilean H1 A (ChH1A), and a Chilean H1 B (ChH1B), which cluster separately on the phylogenetic tree but both of them are classified as “Other-Human-1B.2” according to the new global clade designation^13^; and the A(H1N1)pdm09-like classified as global clade 1A.3.3.2 (Figs. 1 and 2, Supplementary Fig. S1). Fourteen viruses from 10 farms were grouped within cluster ChH1A, 3 viruses from 1 farm were grouped within cluster ChH1B, and 21 viruses from 15 farms were grouped within the A(H1N1)pdm09-like cluster.

Clusters ChH1A and ChH1B were genetically distant from the α (1A.1), β (1A.2), γ (1A.3.2 and 1A.3.3.3), and δ (1B.2.1, 1B.2.2, 1B.2.2.1 and 1B.2.2.2) clades of the North American H1 swine IAVs (Fig. 1, Supplementary Fig. S1 and Table 1). The δ clade 1B.2.1 was the closest North American clade to these Chilean H1 clusters; however, they were over 9.2% distant at the amino acid sequence level. Between δ clade 1B.2.1 and ChH1A, the amino acid identities were 82.5–89.6% and 72.0–90.0% for the HA1 domain and the antigenic sites, respectively; whereas between δ clade 1B.2.1 and ChH1B, amino acid identities were 85.9–90.8% and 72.0–84.0% for the HA1 domain and the antigenic sites, respectively. The amino acid identities between clusters ChH1A and ChH1B were 86.5–87.7% for the HA1 domain, and 78.0–86.0% for the antigenic sites. There was a high variability within cluster ChH1A, compared to cluster ChH1B, with 86.0–100% of amino acid identity in the antigenic sites (Table 1). The main reason for this variability were the strains A/swine/Chile/VN1401-339/2014(H1N2), A/swine/Chile/VN1401-343/2014(H1N2) and A/swine/Chile/VN1401-367/2014(H1N2), which were grouped separately from the rest of ChH1A strains. These strains were on average 6% genetically distant from the others ChH1A strains and 5.6% distant from the center of the cluster (Fig. 2, Supplementary Fig. S1). The most variable antigenic sites among the ChH1A viruses were Ca1 and Cb, with up to 27.3% and 33.3% of amino acid distance, respectively (Supplementary Table S3).

**Table 1:** Comparison based on the percent amino acid identity of the HA1 domain and their antigenic sites between Chilean (ChH1A and ChH1B) and North American (α, β, γ, and δ) H1 swine IAV clusters.

	ChH1A cluster		ChH1B cluster	
	HA1	Antigenic sites	HA1	Antigenic sites
ChH1A cluster	92.6–100	86.0–100	—	—
ChH1B cluster	86.5–87.7	78.0–86.0	98.2–100	96.0–100
α clade 1A.1	68.7–76.1	42.0–54.0	72.4–81.0	40.0–52.0
β clade 1A.2	65.4–72.5	42.0–56.0	69.0–75.2	40.0–52.0
γ clade 1A.3.2 (γ-2)	67.8–73.1	44.0–56.0	71.8–75.2	40.0–54.0
γ clade 1A.3.3.3 (γ)	66.7–72.2	40.0–52.0	71.5–75.5	40.0–52.0
δ clade 1B.2.1 (δ-2)	82.5–89.6	72.0–90.0	85.9–90.8	72.0–84.0
δ clade 1B.2.2 (δ-1)	83.7–89.3	68.0–86.0	87.7–90.8	70.0–84.0
δ clade 1B.2.2.1 (δ-1a)	82.8–87.4	70.0–84.0	85.5–90.2	68.0–78.0
δ clade 1B.2.2.2 (δ-1b)	82.2–86.2	66.0–80.0	85.3–89.6	68.0–78.0

**Figure 3:**
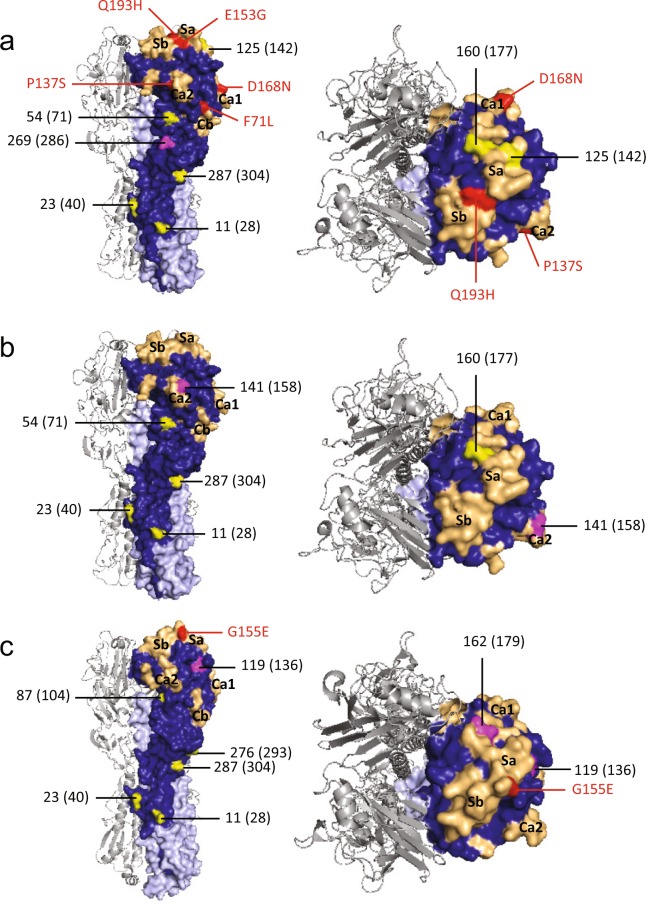
Glycosylation sites and amino acid substitution positions in the HA1 domain contributing to the antigenic diversity of Chilean H1 swine IAVs. Trimeric HA 3D structures with surface representation of one of their monomers, in which HA1 domain is in blue and HA2 domain is in light blue. A side view is shown on the left and a top view is shown on the right. Conserved glycosylation sites present in reference swine IAV vaccine strains are highlighted in yellow; while unique or not-conserved glycosylation sites, which are not present in reference swine IAV strains from current commercial vaccines, are highlighted in magenta. The absolute HA numbering (starting at the first methionine position) are provided in parentheses since some studies have used this nomenclature to designate glycosylation sites. Residues of the antigenic sites (Sa, Sb, Ca1, Ca2 and Cb) are colored light orange, and unique amino acid substitutions in the antigenic sites of antigenic variants are colored red. (**a**) Representative HA of ChH1A cluster using the HA structure of A/Thailand/CU44/2006(H1N1) strain (PDB ID: 4edb). (**b**) Representative HA of ChH1B cluster using the HA structure of the A/Thailand/CU44/2006(H1N1) strain. (**c**) Representative HA of the A(H1N1)pdm09-like cluster was based on the HA structure of the A/California/04/2009(H1N1) strain (PDB ID: 3lzg).

Of interest, the ChH1A IAV strains have an additional glycosylation site at residue 269, which is not present in the ChH1B, North American H1, nor A(H1N1)pdm09-like strains. Moreover, ChH1A viruses have a glycosylation site at residue 125 on antigenic site Sa, which is not present in ChH1B viruses. Compared to the ChH1A cluster, the ChH1B viruses have an additional glycosylation site at residue 141 on antigenic site Ca2, which was also found in 1B.2.1 strains from 2009 onwards. Both ChH1A and ChH1B sequences lack a glycosylation site located at residue 87, which is present in sequences from North American swine H1 strains (Fig. 3a,b, Supplementary Fig. S2a and Supplementary Table S4).

The Chilean and North American A(H1N1)pdm09-like IAV strains were grouped in the same phylogenetic cluster (Fig. 1, Supplementary Fig. S1). The amino acid identities between Chilean A(H1N1)pdm09-like strains and the A(H1N1)pdm09 strain A/California/04/2009(H1N1) used in the current commercial vaccine were 94.2–97.9% for HA1 domain, and 82.0–96.0% for the antigenic sites (Table 2). Some Chilean A(H1N1)pdm09-like strains have additional glycosylation sites at residues 119 (4 out of 21 sequences), near antigenic site Sa, and 162 (1 out of 21 sequences), on antigenic site Sa, which are not present in the A(H1N1)pdm09 A/California/04/2009(H1N1) vaccine strain (Fig. 3c, Supplementary Fig. S2b). Notably, the Chilean A(H1N1)pdm09-like strains were highly variable (Fig. 2), with an amino acid identity ranging from 76.0–100% in antigenic sites (Table 2) and an average genetic distance of 4.7% between strains (Supplementary Table S5). The most variable antigenic sites among the Chilean A(H1N1)pdm09-like viruses were Sa and Ca2, with an amino acid distance of up to 30.8% and 37.5%, respectively (Supplementary Table S3). Of note, in all cases the amino acid identities in antigenic sites were lower than in the complete HA1 region, indicating an increased amino acid substitution rate at these sites.

**Table 2:** Comparison based on the percent amino acid identity of the HA1 domain and their antigenic sites between Chilean A(H1N1)pdm09-like swine IAVs and the commercial pandemic vaccine strain A/California/04/2009(H1N1).

	Chilean A(H1N1)pdm09-like swine IAVs	
	HA1	Antigenic sites
Chilean A(H1N1)pdm09-like swine IAVs	91.4–100	76.0–100
A/California/04/2009(H1N1)	94.2–97.9	82.0–96.0

### Glycosylations in HA reveal a differential evolution of ancestral human IAVs in swine

We analyzed human HA1 sequences to compare the glycosylation pattern found in Chilean swine IAVs since the HA segments of ChH1A and ChH1B viruses were likely introduced from humans in the late 1980s to the early 1990s^24^. Notably, the glycosylation site 141 identified in antigenic site Ca2 of ChH1B viruses was not found in H1 IAV sequences circulating in humans worldwide, analyzed using 8,579 sequences from the last nine decades (1933 to 2017). These results suggest that the glycosylation site 141 was possibly acquired in Chilean pigs after its introduction. In contrast, the glycosylation site 87 (near antigenic site Cb) was found in human HA1 sequences from 1937 to the present, but it was not found in ChH1A and ChH1B viruses, suggesting that this glycosylation disappeared upon circulation in the pigs during the last decades. Moreover, glycosylation site 54 found in both ChH1A and ChH1B viruses was present in human H1 IAVs from 1986, but glycosylation site 269 found in ChH1A viruses disappeared in human H1 viruses from 2000 onwards. Overall, these unique glycosylation patterns seen in the swine strains support the likely introduction dates from humans to swine mentioned above, and also highlight the separate and distinct evolution of these viruses in swine vs. humans.

Even though the introduction of A(H1N1)pdm09 viruses from humans to swine began more recently and occurs regularly^37^, disparate evolutionary patterns in the A(H1N1)pdm09-like swine viruses are also evident. For instance, glycosylation site 119 located near antigenic site Sa of some Chilean swine sequences was not conserved in human A(H1N1)pdm09 sequences and was only found in 9 out of 7,078 (0.1%) sequences analyzed worldwide. In addition, this glycosylation site was not found in Chilean human A(H1N1)pdm09 sequences publicly available between 2009 and 2017.

### Evidence of antigenic variants of Chilean H1 swine IAVs

**Figure 4:**
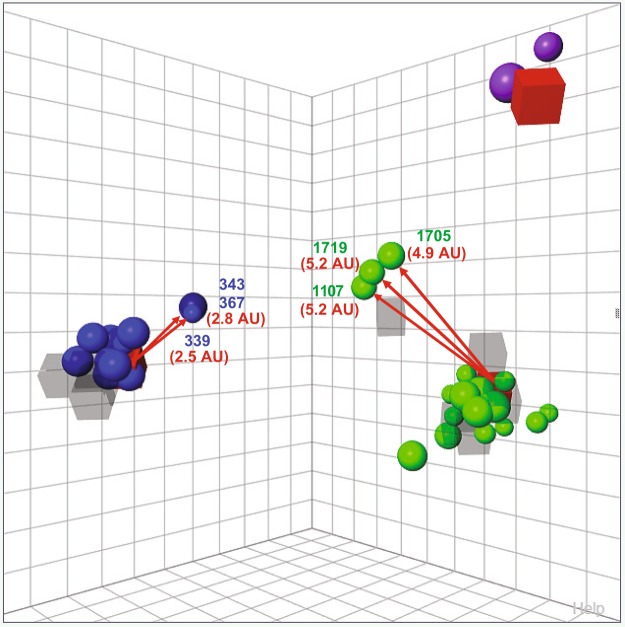
Antigenic diversity of Chilean H1 swine IAVs. A three-dimensional antigenic map was constructed with cross-HI titers among ChH1A, H1ChB and A(H1N1)pdm09-like strains. All axes represent antigenic distance, and the orientation of the map within these axes is free. The spacing between grid lines is 1 unit of antigenic distance (antigenic unit or AU), corresponding to a 2-fold dilution of antiserum in the HI assay. Circles correspond to viruses and squares to antisera analyzed. Antigenic clusters were defined by Ward’s method based on the Euclidean distances among strains. Color represents the antigenic clusters: ChH1A is blue, ChH1B is purple, and A(H1N1)pdm09-like is green. The antisera from the strains A/swine/Chile/VN1401-274/2014(H1N2), A/swine/Chile/VN1401-4/2014(H1N2) and A/swine/Valparaiso/VN1401-559/2014(H1N1), highlighted as a red square, had the broadest cross-reactivity and, therefore, they were the nearest antisera to the center of the antigenic clusters ChH1A, ChH1B and A(H1N1)pdm09-like, respectively. Values in AU and red arrows show the distance between these central antisera and the outlying antigens (antigenic variants) in each cluster: A/swine/Chile/VN1401-339/2014(H1N2) (339), A/swine/Chile/VN1401-343/2014(H1N2) (343) and A/swine/Chile/VN1401-367/2014(H1N2) (367) from ChH1A, and A/swine/Rancagua/VN1401-1107/2015(H1N1) (1107), A/swine/Chillan/VN1401-1705/2015(H1N1) (1705) and A/swine/Chillan/VN1401-1719/2015(H1N1) (1719) from A(H1N1)pdm09-like.

We performed HI assays to define and analyze Chilean swine IAV antigenic clusters. Chilean swine H1 strains were grouped in 3 antigenic clusters, which corresponded with the genetic clusters, ChH1A, ChH1B, and A(H1N1)pdm09-like (Fig. 4). There was no antigenic cross-reactivity between the clusters. The antisera obtained from the strains A/swine/Chile/VN1401-274/2014(H1N2), A/swine/Chile/VN1401-4/2014(H1N2) and A/swine/Valparaiso/VN1401-559/2014(H1N1) had the broadest cross-reactivity and therefore, were the nearest antisera to the center of antigenic clusters ChH1A, ChH1B and A(H1N1)pdm09-like, respectively. Notably, some ChH1A and A(H1N1)pdm09-like strains were antigenically distant from their respective antigenic clusters. The ChH1A strains A/swine/Chile/VN1401-339/2014(H1N2), A/swine/Chile/VN1401-343/2014(H1N2) and A/swine/Chile/VN1401-367/2014(H1N2), isolated from the same farm, were on average 3.1 antigenic units (AU) distant from the other ChH1A strains, and 2.7 AU distant from the central antiserum A/swine/Chile/VN1401-274/2014(H1N2). The rest of ChH1A viruses had on average only 0.95 AU distance between them and thus were considered antigenically similar overall. The A(H1N1)pdm09-like strain A/swine/Rancagua/VN1401-1107/2015(H1N1) was on average 5.9 AU distant from the other Chilean swine A(H1N1)pdm09-like IAVs and 5.2 AU from the central antiserum A/swine/Valparaiso/VN1401-559/2014(H1N1). Similarly, the A(H1N1)pdm09-like strains A/swine/Chillan/VN1401-1705/2015(H1N1) and A/swine/Chillan/VN1401-1719/2015(H1N1), isolated from a different farm, were on average 5.8 AU away from the others Chilean swine A(H1N1)pdm09-like viruses and 4.9 and 5.2 AU, respectively, from the antiserum A/swine/Valparaiso/VN1401-559/2015(H1N1). The strain A/swine/Rancagua/VN1401-1107/2015(H1N1) was antigenically close to the strains A/swine/Chillan/VN1401-1705/2015(H1N1) (1.9 AU) and A/swine/Chillan/VN1401-1719/2015(H1N1) (0.5 AU), despite coming from different farms and different pork production companies that were not only geographically distant but also lacked epidemiological contacts. The rest of the A(H1N1)pdm09-like swine IAVs, without these outliers, had on average 2 AU distance between them (Fig. 4, Supplementary Table S6). These results suggest that these outlier strains are antigenic variants, which had possibly undergone substantial antigenic drift.

### Antigenic variants have unique amino acid substitutions in antigenic sites

We analyzed the aligned amino acid sequences in each cluster in order to identify unique substitutions that could explain the antigenic change in the antigenic variants. The strains A/swine/Chile/VN1401-339/2014(H1N2), A/swine/Chile/VN1401-343/2014(H1N2) and A/swine/Chile/VN1401-367/2014(H1N2) differed from the rest of ChH1A sequences at 17 amino acid positions. Five of these amino acid substitutions were in antigenic sites: E153G in antigenic site Sa, Q193H in antigenic site Sb, D168N in antigenic site Ca1, P137S in antigenic site Ca2, and F71L in antigenic site Cb (Fig. 3a, Table 3). A(H1N1)pdm09-like strains A/swine/Rancagua/VN1401-1107/2015(H1N1), A/swine/Chillan/VN1401-1705/2015(H1N1) and A/swine/Chillan/VN1401-1719/2015(H1N1) had two substitutions in common (R113K and G155E) that differed from the rest of A(H1N1)pdm09-like sequences; however, only G155E was located in an antigenic site (Sa) (Fig. 3c, Table 4). Overall, this suggests that these substitutions could be key in the antigenic drift and evolution of these swine IAVs.

**Table 3:** Presence (+) and absence (−) of unique amino acid substitutions in ChH1A antigenic variants, compared to the rest of ChH1A sequences.

Substitutions	Antigenic site	A/swine/Chile/VN1401–339/2014(H1N2)	A/swine/Chile/VN1401–343/2014(H1N2)	A/swine/Chile/VN1401–367/2014(H1N2)
I19V*	−	+	+	+
I30V*	−	+	+	+
S36N	−	−	−	+
R40K*	−	+	+	+
F71L*	Cb	+	+	+
E120G*	−	+	+	+
V134E*	−	+	+	+
P137S*	Ca2	+	+	+
E153G*	Sa	+	+	+
D168N*	Ca1	+	+	+
E171K*	−	+	+	+
Q193H*	Sb	+	+	+
V202A	−	+	+	−
H205N	Ca1	−	−	+
V220I*	−	+	+	+
D238E*	−	+	+	+
I241T*	−	+	+	+
E273D*	−	+	+	+
N276D*	−	+	+	+
I314M*	−	+	+	+

^*^Amino acid substitutions found in all ChH1A antigenic variants.

**Table 4:** Presence (+) and absence (−) of unique amino acid substitutions in pandemic antigenic variants (A/swine/Rancagua/VN1401-1107/2015(H1N1), A/swine/Chillan/VN1401-1705/2015(H1N1) and A/swine/Chillan/VN1401-1719/2015(H1N1)) compared to the rest of Chilean A(H1N1)pdm09-like sequences.

Substitutions	Antigenic site	A/swine/Rancagua/VN1401-1107/2015(H1N1)	A/swine/Chillan/VN1401-1705/2015(H1N1)	A/swine/Chillan/VN1401-1719/2015(H1N1)
V24I	−	−	+	+
A48S	−	−	+	+
R113K*	−	+	+	+
K/N119S	−	+	−	−
V132L	−	+	−	−
A139D	Ca2	−	+	+
G155E*	Sa	+	+	+
K160M	Sa	−	+	+
S162N	Sa	+	−	−
D168N	Ca1	−	+	+
S183P	−	+	−	−
I/R216K	−	−	+	+
V249I	−	+	−	−
A261T	−	−	+	+
T270A	−	−	+	+
D274N	−	+	−	−
T310A	−	+	−	−

^*^Amino acid substitutions found in all A(H1N1)pdm09-like antigenic variants.

## Discussion

Here, we determined the antigenic diversity of novel H1 IAVs that circulate endemically in Chilean commercial swine farms and identified three genetically and antigenically distinct H1 clusters: ChH1A, ChH1B and A(H1N1)pdm09-like. The ChH1A and A(H1N1)pdm09-like viruses were commonly detected co-circulating concurrently in several farms, while the ChH1B viruses were isolated from a single farm.

The HA sequences from clusters ChH1A and ChH1B were previously classified within the human seasonal lineage Other-Human-1B.2^26^, according to the global swine H1 clade classification^13^. However, although both were of the Other-Human-1B.2 global clade, ChH1A and ChH1B were vastly distinct genetically. The HA segments of these Chilean IAVs were likely introduced into swine from humans in the late 1980s and early 1990s^24^. Hence, these viruses evolved in the swine population for over two decades, and now they are genetically distant from other human IAVs described worldwide^24–26^. Moreover, these Chilean strains have unique glycosylation site patterns, which were not detected in human IAV strains^20^. This reveals the parallel evolution of these ancestral human IAVs in swine, which could represent a public health concern due to the possible lack of human population immunity against these viruses^38,39^. A similar situation was also recently reported in Brazil, where human-origin H1N2 viruses have circulated undetected in swine for more than a decade^40^, some of which have caused zoonotic events^41^.

In Chile, North American commercial vaccines are currently used in some swine farms in an attempt to control the disease. These vaccines contain strains from α clade 1A.1, β clade 1A.2, γ clade 1A.3.3.3, δ clades 1B.2.1 or 1B.2.2, and/or pandemic clade 1A.3.3.2. However, Chilean strains from clusters ChH1A and ChH1B are genetically distant from these North American IAV strains, with different glycosylation patterns and a remarkable variability at antigenic sites. As expected, due to their common and recent introduction from humans-to-swine, the pandemic vaccine strain A/California/04/2009(H1N1) was closely genetically related to Chilean pandemic strains. Nevertheless, we found a high antigenic variability within the Chilean A(H1N1)pdm09-like cluster and some Chilean strains had additional glycosylation sites in or near the antigenic site Sa. This site is one of the most variable antigenic sites among Chilean A(H1N1)pdm09-like viruses, which has been shown to be important for the antigenic evolution of A(H1N1)pdm09-like viruses^42–45^. Of interest, previously it has been shown that most of glycosylation sites acquired in H1 viruses from 1918 to 2009 have been located in or near antigenic site Sa^20^. These findings suggest that site Sa is an important immuno-modulatory site, and that significant structural changes within this site should be particularly considered when evaluating antigenic differences based on HA amino acid sequences. Overall, these results strongly suggest the need to develop swine influenza vaccines with strains that represent the antigenic clusters circulating in Chilean pigs.

There was less amino acid identity in the antigenic sites than in the complete HA1 region, indicating the dominance of non-synonymous substitutions at these sites^46^. Some antigenic variants were identified within clusters ChH1A and A(H1N1)pdm09-like, which had more than 2.4 AU distance compared to the other strains from their respective clusters. It is important to note that a difference higher than 2 AU between circulating and vaccine strains is indicative of a lack of cross-reactivity and thus is one of the accepted criteria for updating the human seasonal IAV vaccine^47^. These antigenic variants that lack cross-reactivity have been shown to have amino acid substitutions in antigenic sites that likely interfere with the immune response mediated by neutralizing antibodies^45,48–50^. Substitutions in all antigenic sites (E153G in Sa, Q193H in Sb, D168N in Ca1, P137S in Ca2, and F71L in Cb) were detected in antigenic variants from ChH1A cluster, whereas only a single substitution in the antigenic site Sa (G155E) was detected in the antigenic variants from the A(H1N1)pdm09-like cluster. The presence of various substitutions in the ChH1A antigenic variants could explain the genetic divergence of these viruses in the phylogenetic tree and genetic map, unlike A(H1N1)pdm09-like antigenic variants, whose antigenic differences were not appreciated in those analyses. Given that a single amino acid substitution can be sufficient to modulate the antigenic properties of HA^20,51,52^, our data confirms the importance to carrying out antigenic analyses in addition to genetic and phylogenetic analyses to fully characterize circulating IAV strains and to evaluate control measures such as vaccination.

Substitutions at the position 71, in the antigenic site Cb, have previously driven antigenic drift of human seasonal^50^ and swine H1 IAVs^49^. The substitutions E153G and G155E found in antigenic variants from the ChH1A and A(H1N1)pdm09-like clusters, respectively, are part of the 151–159 loop (153–157 region), a prominent structure on the top left of the receptor binding site and part of antigenic site Sa^42^. Koel *et al*. showed that several substitutions in this loop cause antigenic escape while retaining the receptor binding capacity and replication fitness^45^. Thus, this loop is considered an important region for antigenic evolution of H1 IAVs and a potential ‘hot spot’ for substitutions that permit immune evasion. However, changes in this region could also emerge after virus propagation in cell culture^42,53,54^. Amino acid substitutions at the position 153 have led to antigenic change in human seasonal^50^ and pandemic H1 IAVs^45^, of which the specific substitution E153G has shown a low impact on antigenicity. These low-impact substitutions are common in the evolution of IAVs and are useful for tracking antigenic evolution^50^. The amino acid substitution G155E has been previously reported as causing a high-impact antigenic change in A(H1N1)pdm09 IAVs^44,45,55,56^. This substitution is analogous to the substitution G158E, which is responsible for major antigenic changes during evolution of H3N2 IAVs^51^.

Amino acid substitutions outside the predicted antigenic sites were also found in the antigenic variants from ChH1A and A(H1N1)pdm09-like clusters. Several amino acid substitutions with antigenic impact, which were not part of these antigenic sites, have been detected in previous studies^44,50^, including the substitution E120G that we identified in the antigenic variants of the ChH1A cluster. Koel *et al*. detected 2 to 3 AU distance between pandemic strains with or without the substitution G155E^45^, whereas we identified more than 5 AU distance between the pandemic antigenic variants and the rest of strains from A(H1N1)pdm09-like cluster. This is noteworthy because the pandemic antigenic variants also have 1 amino acid substitution (R113K) located outside the antigenic sites, which could be of antigenic relevance, reinforcing the need to further characterize the HA of novel H1 IAV strains.

In conclusion, our data suggests that an update of the vaccines used in swine farms in Chile would be necessary to efficiently control the disease due to the existence of novel IAVs. These viruses are genetically distant from other human and swine IAVs described worldwide, including the North American strains currently used in commercial vaccines. An efficient control is imperative, not only to improve production parameters for the swine industry, but also to mitigate and prevent human health risks due to zoonotic events. The methodology used in this study could help identify IAV strains with the broadest cross-reactivity within antigenic clusters, which could then be selected as vaccine candidates. This is the first study addressing the in-depth antigenic characterization of IAVs from swine in Chile, highlighting potential key amino acid substitutions and glycosylation sites capable of modulating the antigenic evolution of these viruses. Further antigenic studies to analyze the evolution of IAV in Chilean swine can be conducted using these strains as well-characterized references. Continuous IAV surveillance and further studies must be carried out to determine if the antigenic variants identified in this study are maintained in the Chilean swine population. Our results describe novel reference virus from South America contributing to global IAV studies in both humans and animals.

## Supplementary information

Supplementary information.

## Data Availability

Nucleic acid sequences generated and/or analyzed during the current study are available in the GenBank repository, https://www.ncbi.nlm.nih.gov/genbank/. The GenBank accession numbers and the remaining data generated or analyzed during this study are included in this published article (and its Supplementary Information Files).
