# Supplementary material for: Antigenic characterization of novel H1 influenza A viruses in swine
Source: Sci Rep. 2020 Mar 11;10:4510. doi: 10.1038/s41598-020-61315-5 (PMC7066140; doi:10.1038/s41598-020-61315-5)
Supplement: Supplementary file 1 — Supplementary information. [file 41598_2020_61315_MOESM1_ESM.pdf]

Antigenic characterization of novel H1 influenza A viruses in swine

Rodrigo Tapia[1], Montserrat Torremorell[2], Marie Culhane[2], Rafael A. Medina[3,4,*], Víctor Neira[1,*]

[1] Departamento de Medicina Preventiva Animal, Facultad de Ciencias Veterinarias y Pecuarias, Universidad de Chile, Santiago, 8820808, Chile.

[2] Department of Veterinary Population Medicine, College of Veterinary Medicine, University of Minnesota, St. Paul, 55108, USA.

[3] Departamento de Enfermedades Infecciosas e Inmunología Pediátrica, Escuela de Medicina, Pontificia Universidad Católica de Chile, Santiago, 8330024, Chile.

[4] Department of Microbiology, Icahn School of Medicine at Mount Sinai, New York, 10029, USA.

*victorneira@u.uchile.cl, rmedinai@uc.cl

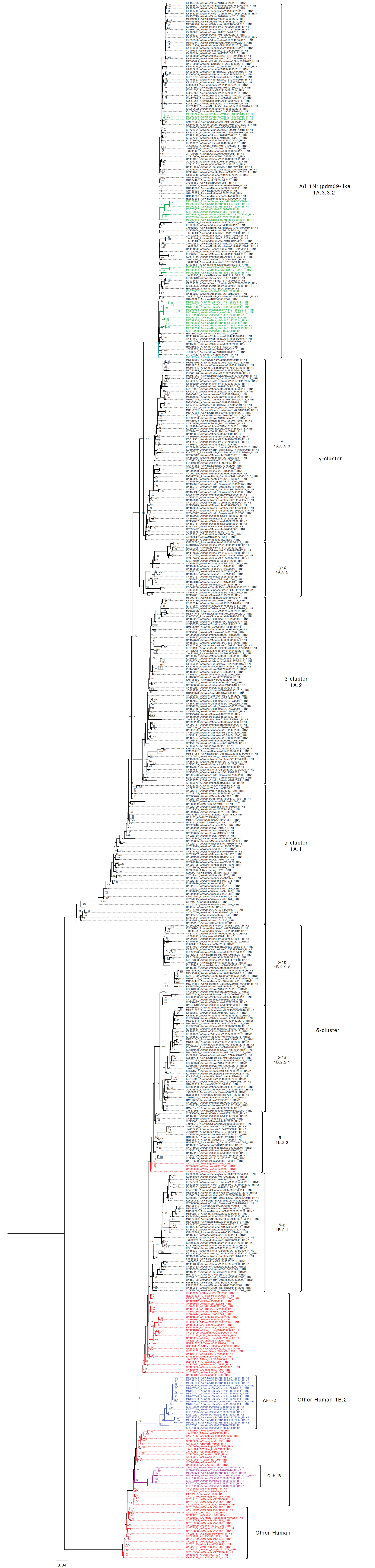
A(H1N1)pdm09-like
1A.3.3.2
γ
1A.3.3.3
γ-cluster
γ-2
1A.3.2
β-cluster
1A.2
α-cluster
1A.1
δ-1b
1B.2.2.2
δ-cluster
δ-1a
1B.2.2.1
δ-1
1B.2.2
δ-2
1B.2.1
ChH1A
Other-Human-1B.2
ChH1B
Other-Human
0.04

Supplementary Figure S1. Phylogenetic tree based on HA1 domain nucleotide sequences of H1 swine IAVs. Phylogenetic tree based on HA1 domain nucleotide sequences of H1 swine IAVs, obtained using the Maximum Likelihood method and General Time Reversible model with a variation rate among sites given by gamma distribution with invariant sites (GTR+G+I). Five hundred eighty-nine sequences were used to construct this phylogenetic tree. Chilean swine IAVs (including reference sequences) are grouped in three phylogenetic clusters: Chilean H1A (ChH1A; blue), Chilean H1B (ChH1B; purple) and pandemic H1N1 2009-like (A(H1N1)pdm09-like; green). Reference sequences from human seasonal IAVs (1977-2008) are in red, and North American H1 swine clusters (α clade 1A.1; β clade 1A.2; γ clades 1A.3.2 and 1A.3.3.3; δ clades 1B.2.1, 1B.2.2, 1B.2.2.1 and 1B.2.2.2; and A(H1N1)pdm09-like clade 1A.3.3.2), used in commercial vaccines, are in black. The pandemic vaccine strain A/California/04/2009(H1N1) is highlighted in cyan.

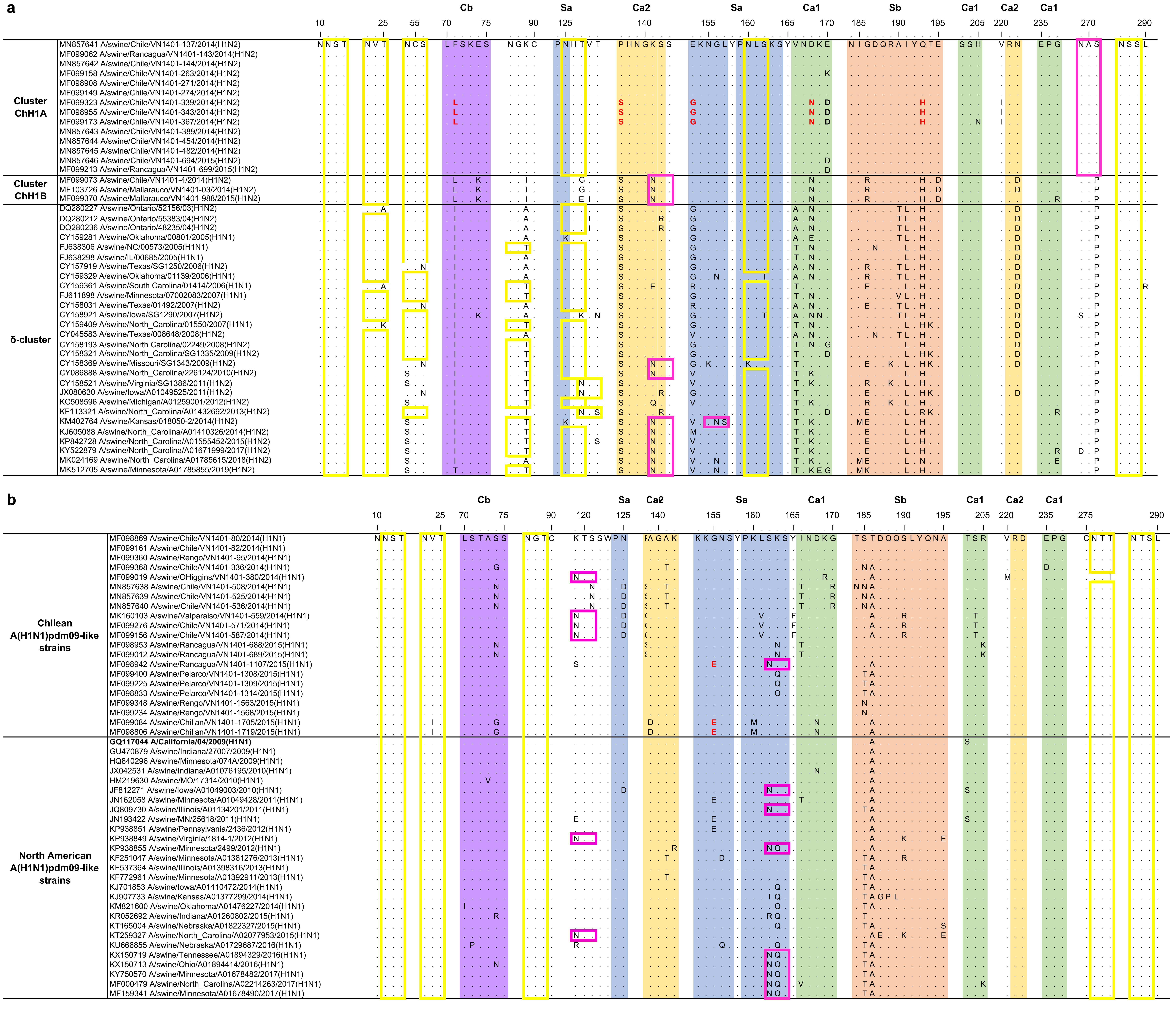
a
Cb
Sa
Ca2
Sa
Ca1
Sb
Ca1
Ca2
Ca1
Cluster ChH1A
MN857641 A/swine/Chile/VN1401-137/2014(H1N2)
MF099062 A/swine/Rancagua/VN1401-143/2014(H1N2)
MN857642 A/swine/Chile/VN1401-144/2014(H1N2)
MF099158 A/swine/Chile/VN1401-263/2014(H1N2)
MF098908 A/swine/Chile/VN1401-271/2014(H1N2)
MF099149 A/swine/Chile/VN1401-274/2014(H1N2)
MF099323 A/swine/Chile/VN1401-339/2014(H1N2)
MF098955 A/swine/Chile/VN1401-343/2014(H1N2)
MF099173 A/swine/Chile/VN1401-367/2014(H1N2)
MN857643 A/swine/Chile/VN1401-389/2014(H1N2)
MN857644 A/swine/Chile/VN1401-454/2014(H1N2)
MN857645 A/swine/Chile/VN1401-482/2014(H1N2)
MN857646 A/swine/Chile/VN1401-694/2015(H1N2)
MF099213 A/swine/Rancagua/VN1401-699/2015(H1N2)
Cluster ChH1B
MF099073 A/swine/Chile/VN1401-4/2014(H1N2)
MF103726 A/swine/Mallarauco/VN1401-03/2014(H1N2)
MF099370 A/swine/Mallarauco/VN1401-988/2015(H1N2)
δ-cluster
DQ280227 A/swine/Ontario/52156/03(H1N2)
DQ280212 A/swine/Ontario/55383/04(H1N2)
DQ280236 A/swine/Ontario/48235/04(H1N2)
CY159281 A/swine/Oklahoma/00801/2005(H1N1)
FJ638306 A/swine/NC/00573/2005(H1N1)
FJ638298 A/swine/IL/00685/2005(H1N1)
CY157919 A/swine/Texas/SG1250/2006(H1N2)
CY159329 A/swine/Oklahoma/01139/2006(H1N1)
CY159361 A/swine/South Carolina/01414/2006(H1N1)
FJ611898 A/swine/Minnesota/07002083/2007(H1N1)
CY158031 A/swine/Texas/01492/2007(H1N1)
CY158921 A/swine/Iowa/SG1290/2007(H1N2)
CY159409 A/swine/North_Carolina/01550/2007(H1N1)
CY045583 A/swine/Texas/008648/2008(H1N2)
CY158193 A/swine/North Carolina/02249/2008(H1N2)
CY158321 A/swine/North_Carolina/SG1335/2009(H1N2)
CY158369 A/swine/Missouri/SG1343/2009(H1N2)
CY086888 A/swine/North_Carolina/226124/2010(H1N2)
CY158521 A/swine/Virginia/SG1386/2011(H1N2)
JX080630 A/swine/Iowa/A01049525/2011(H1N2)
KC508596 A/swine/Michigan/A01259001/2012(H1N2)
KF113321 A/swine/North_Carolina/A01432692/2013(H1N2)
KM402764 A/swine/Kansas/018050-2/2014(H1N2)
KJ605088 A/swine/North_Carolina/A01410326/2014(H1N2)
KP842728 A/swine/North_Carolina/A01555452/2015(H1N2)
KY522879 A/swine/North_Carolina/A01671999/2017(H1N2)
MK024169 A/swine/North_Carolina/A01785615/2018(H1N2)
MK512705 A/swine/Minnesota/A01785855/2019(H1N2)
b
Cb
Sa
Ca2
Sa
Ca1
Sb
Ca1
Ca2
Ca1
Chilean A(H1N1)pdm09-like strains
MF098869 A/swine/Chile/VN1401-80/2014(H1N1)
MF099161 A/swine/Chile/VN1401-82/2014(H1N1)
MF099360 A/swine/Rengo/VN1401-95/2014(H1N1)
MF099368 A/swine/Chile/VN1401-336/2014(H1N1)
MF099019 A/swine/OHiggins/VN1401-380/2014(H1N1)
MN857638 A/swine/Chile/VN1401-508/2014(H1N1)
MN857639 A/swine/Chile/VN1401-525/2014(H1N1)
MN857640 A/swine/Chile/VN1401-536/2014(H1N1)
MK160103 A/swine/Valparaiso/VN1401-559/2014(H1N1)
MF099276 A/swine/Chile/VN1401-571/2014(H1N1)
MF099156 A/swine/Chile/VN1401-587/2014(H1N1)
MF098953 A/swine/Rancagua/VN1401-688/2015(H1N1)
MF099012 A/swine/Rancagua/VN1401-689/2015(H1N1)
MF098942 A/swine/Rancagua/VN1401-1107/2015(H1N1)
MF099400 A/swine/Pelarco/VN1401-1308/2015(H1N1)
MF099225 A/swine/Pelarco/VN1401-1309/2015(H1N1)
MF098833 A/swine/Pelarco/VN1401-1314/2015(H1N1)
MF099348 A/swine/Rengo/VN1401-1563/2015(H1N1)
MF099234 A/swine/Rengo/VN1401-1568/2015(H1N1)
MF099084 A/swine/Chillan/VN1401-1705/2015(H1N1)
MF098806 A/swine/Chillan/VN1401-1719/2015(H1N1)
North American A(H1N1)pdm09-like strains
GQ117044 A/California/04/2009(H1N1)
GU470879 A/swine/Indiana/27007/2009(H1N1)
HQ840296 A/swine/Minnesota/074A/2009(H1N1)
JX042531 A/swine/Indiana/A01076195/2010(H1N1)
HM219630 A/swine/MO/17314/2010(H1N1)
JF812271 A/swine/Iowa/A01049003/2010(H1N1)
JN162058 A/swine/Minnesota/A01049428/2011(H1N1)
JQ809730 A/swine/Illinois/A01134201/2011(H1N1)
JN193422 A/swine/MN/25618/2011(H1N1)
KP938851 A/swine/Pennsylvania/2436/2012(H1N1)
KP938849 A/swine/Virginia/1814-1/2012(H1N1)
KP938855 A/swine/Minnesota/2499/2012(H1N1)
KF251047 A/swine/Minnesota/A01381276/2013(H1N1)
KF537364 A/swine/Illinois/A01398316/2013(H1N1)
KF772961 A/swine/Minnesota/A01392911/2013(H1N1)
KJ701853 A/swine/Iowa/A01410472/2014(H1N1)
KJ907733 A/swine/Kansas/A01377299/2014(H1N1)
KM821600 A/swine/Oklahoma/A01476227/2014(H1N1)
KR052692 A/swine/Indiana/A01260802/2015(H1N1)
KT165004 A/swine/Nebraska/A01822327/2015(H1N1)
KT259327 A/swine/North_Carolina/A02077953/2015(H1N1)
KU666855 A/swine/Nebraska/A01729687/2016(H1N1)
KX150719 A/swine/Tennessee/A01894329/2016(H1N1)
KX150713 A/swine/Ohio/A01894414/2016(H1N1)
KY750570 A/swine/Minnesota/A01678482/2017(H1N1)
MF000479 A/swine/North_Carolina/A02214263/2017(H1N1)
MF159341 A/swine/Minnesota/A01678490/2017(H1N1)

Supplementary Figure S2. Chilean swine IAVs have differences in the antigenic sites and glycosylation patterns compared to North American swine IAVs. Amino acid alignment of HA1 domain sequences at antigenic and glycosylation sites. Colored shading represents antigenic sites listed on top, according to Gerhard *et al.*[17] and Caton *et al.*[18]. Yellow boxes represent conserved glycosylation sites and magenta boxes represent non-conserved glycosylation sites. Unique amino acid substitutions in the antigenic sites of antigenic variants are highlighted in red. (a) Comparison among strains from ChH1A, ChH1B, and δ (clades 1B.2.1 and 1B.2.2) clusters. (b) Comparison among Chilean and North American A(H1N1)pdm09-like swine IAV strains. The commercial vaccine strain is highlighted in bold.

Supplementary Table S1. Number of samples and sequenced isolates of the subtype H1 obtained from commercial swine farms in Chile. Companies were named by a code (A-V).

No.	Company	No. farms	No. samples	No. sequenced isolates
1	A	3	219	9
2	B	1	64	0
3	C	1	35	0
4	D	1	75	0
5	E	1	34	0
6	F	1	48	4
7	G	1	90	5
8	H	1	40	0
9	I	1	36	0
10	J	12	285	12
11	K	1	56	0
12	L	2	167	5
13	M	1	133	1
14	N	1	18	0
15	O	1	35	0
16	P	1	4	0
17	Q	1	29	0
18	R	1	37	0
19	S	1	38	0
20	T	1	35	0
21	U	1	84	0
22	V	1	118	2
**Total**		**36**	**1680**	**38**

Supplementary Table S2. GenBank accession numbers of HA gene sequences of the Chilean swine IAVs used in this study.

Strain name	GenBank accession no.	Reference
A/swine/Chile/VN1401-137/2014(H1N2)	MN857641	First reported in this study
A/swine/Rancagua/VN1401-143/2014(H1N2)	MF099062	First reported in this study
A/swine/Chile/VN1401-144/2014(H1N2)	MN857642	First reported in this study
A/swine/Chile/VN1401-263/2014(H1N2)	MF099158	First reported in this study
A/swine/Chile/VN1401-271/2014(H1N2)	MF098908	First reported in this study
A/swine/Chile/VN1401-274/2014(H1N2)	MF099149	Tapia et al., 2018[26]
A/swine/Chile/VN1401-339/2014(H1N2)	MF099323	First reported in this study
A/swine/Chile/VN1401-343/2014(H1N2)	MF098955	First reported in this study
A/swine/Chile/VN1401-367/2014(H1N2)	MF099173	First reported in this study
A/swine/Chile/VN1401-389/2014(H1N2)	MN857643	First reported in this study
A/swine/Chile/VN1401-454/2014(H1N2)	MN857644	First reported in this study
A/swine/Chile/VN1401-482/2014(H1N2)	MN857645	First reported in this study
A/swine/Chile/VN1401-694/2015(H1N2)	MN857646	First reported in this study
A/swine/Rancagua/VN1401-699/2015(H1N2)	MF099213	First reported in this study
A/swine/Mallarauco/VN1401-03/2014(H1N2)	MF103726	First reported in this study
A/swine/Chile/VN1401-4/2014(H1N2)	MF099073	Tapia et al., 2018[26]
A/swine/Mallarauco/VN1401-988/2015(H1N2)	MF099370	First reported in this study
A/swine/Chile/VN1401-80/2014(H1N1)	MF098869	First reported in this study
A/swine/Chile/VN1401-82/2014(H1N1)	MF099161	First reported in this study
A/swine/Rengo/VN1401-95/2014(H1N1)	MF099360	First reported in this study
A/swine/Chile/VN1401-336/2014(H1N1)	MF099368	First reported in this study
A/swine/O'Higgins/VN1401-380/2014(H1N1)	MF099019	First reported in this study
A/swine/Chile/VN1401-508/2014(H1N1)	MN857638	First reported in this study
A/swine/Chile/VN1401-525/2014(H1N1)	MN857639	First reported in this study
A/swine/Chile/VN1401-536/2014(H1N1)	MN857640	First reported in this study
A/swine/Valparaiso/VN1401-559/2014(H1N1)	MK160103	Tapia et al., 2018[26]
A/swine/Chile/VN1401-571/2014(H1N1)	MF099276	First reported in this study
A/swine/Chile/VN1401-587/2014(H1N1)	MF099156	First reported in this study
A/swine/Rancagua/VN1401-688/2015(H1N1)	MF098953	First reported in this study
A/swine/Rancagua/VN1401-689/2015(H1N1)	MF099012	First reported in this study
A/swine/Rancagua/VN1401-1107/2015(H1N1)	MF098942	First reported in this study
A/swine/Pelarco/VN1401-1308/2015(H1N1)	MF099400	First reported in this study
A/swine/Pelarco/VN1401-1309/2015(H1N1)	MF099225	First reported in this study
A/swine/Pelarco/VN1401-1314/2015(H1N1)	MF098833	First reported in this study
A/swine/Rengo/VN1401-1563/2015(H1N1)	MF099348	First reported in this study
A/swine/Rengo/VN1401-1568/2015(H1N1)	MF099234	First reported in this study
A/swine/Chillan/VN1401-1705/2015(H1N1)	MF099084	First reported in this study
A/swine/Chillan/VN1401-1719/2015(H1N1)	MF098806	First reported in this study

Supplementary Table S3. Amino acid distance (%) in antigenic sites within Chilean H1 swine IAV clusters.

Clusters	Antigenic sites				
	Sa	Sb	Ca1	Ca2	Cb
**ChH1A**	0-7.7	0-8.3	0-27.3	0-12.5	0-33.3
**ChH1B**	0	0	0-9.1	0-12.5	0
**A(H1N1)pdm09-like**	0-30.8	0-25.0	0-27.3	0-37.5	0-16.7

Supplementary Table S4. Presence (+)/absence (-) of glycosylation sites found in HA1 domain sequences from North American H1 swine IAV clusters.

Clusters	Glycosylation sites															
	11	23	54	87	119	125	127	141	155	160	162	168	184	274	276	287
**α-cluster**	+	+	-	+	+	-	-	-	-	-	+	-	-	-	+	+
**β-cluster**	+	+	-	+	-	-	-	-	-	-	-	+	+	+	+	+
**γ-cluster**	+	+	-	+	+	-	-	-	-	-	+	-	-	-	+	+
**δ-cluster**	+	+	+	+	-	+	+	+	+	+	-	-	-	-	-	+

Supplementary Table S5. Dissimilarity matrix with the percent of distance among HA1 amino acid sequences used to construct the genetic map.

	1	2	3	4	5	6	7	8	9	10	11	12	13	14	15	16	17	18	19	20	21	22	23	24	25	26	27	28	29	30	31	32	33	34	35	36	37	38
1. A/swine/Chile/137/2014(H1N2)	0.0	0.0	0.3	0.9	0.9	0.9	6.8	6.8	7.1	0.9	0.9	1.2	0.9	0.9	13.4	13.4	13.4	32.9	32.6	32.6	32.6	32.9	32.6	32.9	32.9	35.0	35.0	35.0	32.9	32.9	34.1	33.5	33.5	33.5	32.9	32.9	33.2	33.5
2. A/swine/Rancagua/VN1401-143/2014(H1N2)	0.0	0.0	0.3	0.9	0.9	0.9	6.8	6.8	7.1	0.9	0.9	1.2	0.9	0.9	13.4	13.4	13.4	32.9	32.6	32.6	32.6	32.9	32.6	32.9	32.9	35.0	35.0	35.0	32.9	32.9	34.1	33.5	33.5	33.5	32.9	32.9	33.2	33.5
3. A/swine/Chile/VN1401-144/2014(H1N2)	0.3	0.3	0.0	0.6	0.6	0.6	6.5	6.5	6.8	0.6	0.6	0.9	0.6	0.6	13.1	13.1	13.1	32.6	32.3	32.3	32.3	32.6	32.3	32.6	32.6	34.7	34.7	34.7	32.6	32.6	33.8	33.2	33.2	33.2	32.6	32.6	32.9	33.2
4. A/swine/Chile/VN1401-263/2014(H1N2)	0.9	0.9	0.6	0.0	0.6	0.6	6.8	6.8	7.1	1.2	1.2	1.5	0.3	0.3	13.6	13.6	13.6	32.3	32.0	32.0	32.0	32.3	32.0	32.3	32.3	34.4	34.4	34.4	32.3	32.3	33.5	32.9	32.9	32.9	32.3	32.3	32.6	32.9
5. A/swine/Chile/VN1401-271/2014(H1N2)	0.9	0.9	0.6	0.6	0.0	0.0	7.1	7.1	7.4	1.2	1.2	1.5	0.6	0.6	13.6	13.6	13.6	32.0	31.8	31.8	31.8	32.0	31.8	32.0	32.0	34.1	34.1	34.1	32.0	32.0	33.2	32.6	32.6	32.6	32.0	32.0	32.3	32.6
6. A/swine/Chile/VN1401-274/2014(H1N2)	0.9	0.9	0.6	0.6	0.0	0.0	7.1	7.1	7.4	1.2	1.2	1.5	0.6	0.6	13.6	13.6	13.6	32.0	31.8	31.8	31.8	32.0	31.8	32.0	32.0	34.1	34.1	34.1	32.0	32.0	33.2	32.6	32.6	32.6	32.0	32.0	32.3	32.6
7. A/swine/Chile/VN1401-339/2014(H1N2)	6.8	6.8	6.5	6.8	7.1	7.1	0.0	0.0	0.9	6.5	6.8	7.1	6.5	6.5	12.5	12.5	12.5	32.0	31.8	31.8	31.8	31.8	31.5	31.8	31.8	34.1	34.1	34.1	32.3	32.3	33.2	32.6	32.6	32.6	32.0	32.0	31.8	32.0
8. A/swine/Chile/VN1401-343/2014(H1N2)	6.8	6.8	6.5	6.8	7.1	7.1	0.0	0.0	0.9	6.5	6.8	7.1	6.5	6.5	12.5	12.5	12.5	32.0	31.8	31.8	31.8	31.8	31.5	31.8	31.8	34.1	34.1	34.1	32.3	32.3	33.2	32.6	32.6	32.6	32.0	32.0	31.8	32.0
9. A/swine/Chile/VN1401-367/2014(H1N2)	7.1	7.1	6.8	7.1	7.4	7.4	0.9	0.9	0.0	6.8	7.1	7.4	6.8	6.8	12.8	12.8	12.8	32.0	31.8	31.8	31.8	31.8	31.5	31.8	31.8	34.1	34.1	34.1	32.3	32.3	33.2	32.6	32.6	32.6	32.0	32.0	31.8	32.0
10. A/swine/Chile/389/2014(H1N2)	0.9	0.9	0.6	1.2	1.2	1.2	6.5	6.5	6.8	0.0	1.2	1.5	1.2	1.2	13.1	13.1	13.1	32.0	31.8	31.8	31.8	32.6	31.8	32.0	32.0	34.1	34.1	34.1	32.0	32.0	33.2	32.6	32.6	32.6	32.0	32.0	32.3	32.6
11. A/swine/Chile/454/2014(H1N2)	0.9	0.9	0.6	1.2	1.2	1.2	6.8	6.8	7.1	1.2	0.0	0.3	1.2	1.2	13.4	13.4	13.4	32.3	32.0	32.0	32.0	32.3	32.0	32.3	32.3	34.4	34.4	34.4	32.3	32.3	33.5	32.9	32.9	32.9	32.3	32.3	32.6	32.9
12. A/swine/Chile/482/2014(H1N2)	1.2	1.2	0.9	1.5	1.5	1.5	7.1	7.1	7.4	1.5	0.3	0.0	1.5	1.5	13.6	13.6	13.6	32.3	32.0	32.0	32.0	32.3	32.0	32.3	32.3	34.4	34.4	34.4	32.3	32.3	33.5	32.9	32.9	32.9	32.3	32.3	32.6	32.9
13. A/swine/Chile/694/2015(H1N2)	0.9	0.9	0.6	0.3	0.6	0.6	6.5	6.5	6.8	1.2	1.2	1.5	0.0	0.0	13.6	13.6	13.6	32.3	32.0	32.0	32.0	32.3	32.0	32.3	32.3	34.4	34.4	34.4	32.3	32.3	33.5	32.9	32.9	32.9	32.3	32.3	32.6	32.9
14. A/swine/Rancagua/VN1401-699/2015(H1N2)	0.9	0.9	0.6	0.3	0.6	0.6	6.5	6.5	6.8	1.2	1.2	1.5	0.0	0.0	13.6	13.6	13.6	32.3	32.0	32.0	32.0	32.3	32.0	32.3	32.3	34.4	34.4	34.4	32.3	32.3	33.5	32.9	32.9	32.9	32.3	32.3	32.6	32.9
15. A/swine/Mallarauco/VN1401-03/2014(H1N2)	13.4	13.4	13.1	13.6	13.6	13.6	12.5	12.5	12.8	13.1	13.4	13.6	13.6	13.6	0.0	0.0	2.1	28.2	27.9	27.9	28.5	28.2	27.6	27.9	27.9	30.0	30.0	30.0	28.5	28.5	29.7	28.5	28.5	28.5	28.2	28.2	27.6	27.9
16. A/swine/Chile/VN1401-4/2014(H1N2)	13.4	13.4	13.1	13.6	13.6	13.6	12.5	12.5	12.8	13.1	13.4	13.6	13.6	13.6	0.0	0.0	2.1	28.2	27.9	27.9	28.5	28.2	27.6	27.9	27.9	30.0	30.0	30.0	28.5	28.5	29.7	28.5	28.5	28.5	28.2	28.2	27.6	27.9
17. A/swine/Mallarauco/VN1401-988/2015(H1N2)	13.4	13.4	13.1	13.6	13.6	13.6	12.5	12.5	12.8	13.1	13.4	13.6	13.6	13.6	2.1	2.1	0.0	28.8	28.5	28.5	29.1	28.8	28.2	28.5	28.5	30.6	30.6	30.6	29.1	29.1	30.3	29.1	29.1	29.1	28.8	28.8	28.5	28.8
18. A/swine/Chile/VN1401-80/2014(H1N1)	32.9	32.9	32.6	32.3	32.0	32.0	32.0	32.0	32.0	32.0	32.3	32.3	32.3	32.3	28.2	28.2	28.8	0.0	0.3	0.3	3.3	4.2	4.5	4.5	4.2	5.6	5.6	5.6	6.2	6.2	4.7	2.7	2.7	2.7	0.9	0.9	5.0	5.3
19. A/swine/Chile/VN1401-82/2014(H1N1)	32.6	32.6	32.3	32.0	31.8	31.8	31.8	31.8	31.8	31.8	32.0	32.0	32.0	32.0	27.9	27.9	28.5	0.3	0.0	0.0	3.0	3.9	4.2	4.2	3.9	5.3	5.3	5.3	5.9	5.9	4.5	2.4	2.4	2.4	0.6	0.6	4.7	5.0
20. A/swine/Rengo/VN1401-95/2014(H1N1)	32.6	32.6	32.3	32.0	31.8	31.8	31.8	31.8	31.8	31.8	32.0	32.0	32.0	32.0	27.9	27.9	28.5	0.3	0.0	0.0	3.0	3.9	4.2	4.2	3.9	5.3	5.3	5.3	5.9	5.9	4.5	2.4	2.4	2.4	0.6	0.6	4.7	5.0
21. A/swine/Chile/VN1401-336/2014(H1N1)	32.6	32.6	32.3	32.0	31.8	31.8	31.8	31.8	31.8	31.8	32.0	32.0	32.0	32.0	28.5	28.5	29.1	3.3	3.0	3.0	0.0	5.0	3.9	3.9	3.6	6.5	6.5	6.5	6.8	6.8	5.6	3.3	3.3	3.3	3.0	3.0	4.7	5.0
22. A/swine/O'Higgins/VN1401-380/2014(H1N1)	32.9	32.9	32.6	32.3	32.0	32.0	31.8	31.8	31.8	32.6	32.3	32.3	32.3	32.3	28.2	28.2	28.8	4.2	3.9	3.9	5.0	0.0	6.2	6.2	5.9	6.2	6.2	6.2	8.6	8.6	6.2	4.5	4.5	4.5	4.5	4.5	6.8	7.1
23. A/swine/Chile/VN1401-508/2014(H1N1)	32.6	32.6	32.3	32.0	31.8	31.8	31.5	31.5	31.5	31.8	32.0	32.0	32.0	32.0	27.6	27.6	28.2	4.5	4.2	4.2	3.9	6.2	0.0	0.6	0.3	6.2	6.2	6.2	5.9	5.9	6.8	4.5	4.5	4.5	4.2	4.2	6.8	7.1
24. A/swine/Chile/VN1401-525/2014(H1N1)	32.9	32.9	32.6	32.3	32.0	32.0	31.8	31.8	31.8	32.0	32.3	32.3	32.3	32.3	27.9	27.9	28.5	4.5	4.2	4.2	3.9	6.2	0.6	0.0	0.3	6.2	6.2	6.2	5.9	5.9	6.8	4.5	4.5	4.5	4.2	4.2	6.8	7.1
25. A/swine/Chile/VN1401-536/2014(H1N1)	32.9	32.9	32.6	32.3	32.0	32.0	31.8	31.8	31.8	32.0	32.3	32.3	32.3	32.3	27.9	27.9	28.5	4.2	3.9	3.9	3.6	5.9	0.3	0.3	0.0	5.9	5.9	5.9	5.6	5.6	6.5	4.2	4.2	4.2	3.9	3.9	6.5	6.8
26. A/swine/Valparaiso/VN1401-559/2014(H1N1)	35.0	35.0	34.7	34.4	34.1	34.1	34.1	34.1	34.1	34.1	34.4	34.4	34.4	34.4	30.0	30.0	30.6	5.6	5.3	5.3	6.5	6.2	6.2	6.2	5.9	0.0	0.0	0.0	9.2	9.2	7.7	5.0	5.0	5.0	5.9	5.9	8.3	8.3
27. A/swine/Chile/VN1401-571/2014(H1N1)	35.0	35.0	34.7	34.4	34.1	34.1	34.1	34.1	34.1	34.1	34.4	34.4	34.4	34.4	30.0	30.0	30.6	5.6	5.3	5.3	6.5	6.2	6.2	6.2	5.9	0.0	0.0	0.0	9.2	9.2	7.7	5.0	5.0	5.0	5.9	5.9	8.3	8.3
28. A/swine/Chile/VN1401-587/2014(H1N1)	35.0	35.0	34.7	34.4	34.1	34.1	34.1	34.1	34.1	34.1	34.4	34.4	34.4	34.4	30.0	30.0	30.6	5.6	5.3	5.3	6.5	6.2	6.2	6.2	5.9	0.0	0.0	0.0	9.2	9.2	7.7	5.0	5.0	5.0	5.9	5.9	8.3	8.3
29. A/swine/Rancagua/VN1401-688/2015(H1N1)	32.9	32.9	32.6	32.3	32.0	32.0	32.3	32.3	32.3	32.0	32.3	32.3	32.3	32.3	28.5	28.5	29.1	6.2	5.9	5.9	6.8	8.6	5.9	5.9	5.6	9.2	9.2	9.2	0.0	0.0	8.0	6.8	6.8	6.8	6.5	6.5	8.9	9.2
30. A/swine/Rancagua/VN1401-689/2015(H1N1)	32.9	32.9	32.6	32.3	32.0	32.0	32.3	32.3	32.3	32.0	32.3	32.3	32.3	32.3	28.5	28.5	29.1	6.2	5.9	5.9	6.8	8.6	5.9	5.9	5.6	9.2	9.2	9.2	0.0	0.0	8.0	6.8	6.8	6.8	6.5	6.5	8.9	9.2
31. A/swine/Rancagua/VN1401-1107/2015(H1N1)	34.1	34.1	33.8	33.5	33.2	33.2	33.2	33.2	33.2	33.2	33.5	33.5	33.5	33.5	29.7	29.7	30.3	4.7	4.5	4.5	5.6	6.2	6.8	6.8	6.5	7.7	7.7	7.7	8.0	8.0	0.0	5.0	5.0	5.0	5.0	5.0	5.9	6.2
32. A/swine/Pelarco/VN1401-1308/2015(H1N1)	33.5	33.5	33.2	32.9	32.6	32.6	32.6	32.6	32.6	32.6	32.9	32.9	32.9	32.9	28.5	28.5	29.1	2.7	2.4	2.4	3.3	4.5	4.5	4.5	4.2	5.0	5.0	5.0	6.8	6.8	5.0	0.0	0.0	0.0	2.7	2.7	5.3	5.6
33. A/swine/Pelarco/VN1401-1309/2015(H1N1)	33.5	33.5	33.2	32.9	32.6	32.6	32.6	32.6	32.6	32.6	32.9	32.9	32.9	32.9	28.5	28.5	29.1	2.7	2.4	2.4	3.3	4.5	4.5	4.5	4.2	5.0	5.0	5.0	6.8	6.8	5.0	0.0	0.0	0.0	2.7	2.7	5.3	5.6
34. A/swine/Pelarco/VN1401-1314/2015(H1N1)	33.5	33.5	33.2	32.9	32.6	32.6	32.6	32.6	32.6	32.6	32.9	32.9	32.9	32.9	28.5	28.5	29.1	2.7	2.4	2.4	3.3	4.5	4.5	4.5	4.2	5.0	5.0	5.0	6.8	6.8	5.0	0.0	0.0	0.0	2.7	2.7	5.3	5.6
35. A/swine/Rengo/VN1401-1563/2015(H1N1)	32.9	32.9	32.6	32.3	32.0	32.0	32.0	32.0	32.0	32.0	32.3	32.3	32.3	32.3	28.2	28.2	28.8	0.9	0.6	0.6	3.0	4.5	4.2	4.2	3.9	5.9	5.9	5.9	6.5	6.5	5.0	2.7	2.7	2.7	0.0	0.0	5.3	5.6
36. A/swine/Rengo/VN1401-1568/2015(H1N1)	32.9	32.9	32.6	32.3	32.0	32.0	32.0	32.0	32.0	32.0	32.3	32.3	32.3	32.3	28.2	28.2	28.8	0.9	0.6	0.6	3.0	4.5	4.2	4.2	3.9	5.9	5.9	5.9	6.5	6.5	5.0	2.7	2.7	2.7	0.0	0.0	5.3	5.6
37. A/swine/Chillan/VN1401-1705/2015(H1N1)	33.2	33.2	32.9	32.6	32.3	32.3	31.8	31.8	31.8	32.3	32.6	32.6	32.6	32.6	27.6	27.6	28.5	5.0	4.7	4.7	4.7	6.8	6.8	6.8	6.5	8.3	8.3	8.3	8.9	8.9	5.9	5.3	5.3	5.3	5.3	5.3	0.0	0.3
38. A/swine/Chillan/VN1401-1719/2015(H1N1)	33.5	33.5	33.2	32.9	32.6	32.6	32.0	32.0	32.0	32.6	32.9	32.9	32.9	32.9	27.9	27.9	28.8	5.3	5.0	5.0	5.0	7.1	7.1	7.1	6.8	8.3	8.3	8.3	9.2	9.2	6.2	5.6	5.6	5.6	5.6	5.6	0.3	0.0

Supplementary Table S6. Euclidean distance matrix with the antigenic units (AU) among strains used to construct the antigenic map.

	1	2	3	4	5	6	7	8	9	10	11	12	13	14	15	16	17	18	19	20	21	22	23	24	25	26	27	28	29	30	31	32	33	34	35	36	37	38
1. A/swine/Chile/137/2014(H1N2)	0.0	1.8	1.9	0.9	0.9	0.6	3.5	3.8	3.8	2.4	1.0	0.8	0.9	0.9	8.2	8.2	9.1	9.8	10.1	9.9	9.7	10.7	11.0	11.4	11.8	13.3	13.1	13.7	10.9	10.3	10.7	9.8	10.1	9.9	10.2	12.9	8.8	10.2
2. A/swine/Rancagua/VN1401-143/2014(H1N2)	1.8	0.0	0.8	1.0	0.9	1.8	2.7	3.1	3.2	0.6	1.0	1.0	1.0	0.9	10.0	10.0	10.9	11.0	11.3	11.3	11.0	11.9	12.1	12.6	12.9	14.3	14.2	14.8	12.3	11.8	11.1	11.1	11.3	11.3	11.5	14.1	9.4	10.6
3. A/swine/Chile/VN1401-144/2014(H1N2)	1.9	0.8	0.0	1.3	1.2	2.1	3.5	3.9	4.0	0.9	1.4	1.2	1.3	1.2	9.7	9.7	10.7	11.5	11.8	11.7	11.4	12.4	12.6	13.1	13.4	14.9	14.7	15.3	12.7	12.1	11.8	11.5	11.8	11.7	11.9	14.6	10.0	11.3
4. A/swine/Chile/VN1401-263/2014(H1N2)	0.9	1.0	1.3	0.0	0.1	0.8	2.9	3.2	3.2	1.6	0.2	0.2	0.0	0.1	9.0	9.1	10.0	10.2	10.5	10.4	10.1	11.1	11.3	11.8	12.1	13.6	13.4	14.0	11.4	10.9	10.6	10.3	10.5	10.4	10.7	13.3	8.9	10.1
5. A/swine/Chile/VN1401-271/2014(H1N2)	0.9	0.9	1.2	0.1	0.0	0.9	2.8	3.2	3.2	1.5	0.2	0.2	0.1	0.0	9.1	9.2	10.1	10.3	10.5	10.5	10.2	11.2	11.3	11.8	12.2	13.6	13.5	14.1	11.5	10.9	10.7	10.3	10.5	10.5	10.7	13.3	8.9	10.2
6. A/swine/Chile/VN1401-274/2014(H1N2)	0.6	1.8	2.1	0.8	0.9	0.0	3.0	3.2	3.2	2.4	0.8	0.9	0.8	0.9	8.4	8.5	9.4	9.4	9.7	9.6	9.3	10.3	10.5	11.0	11.4	12.8	12.7	13.3	10.6	10.0	10.1	9.5	9.7	9.6	9.9	12.5	8.3	9.6
7. A/swine/Chile/VN1401-339/2014(H1N2)	3.5	2.7	3.5	2.9	2.8	3.0	0.0	0.5	0.6	2.9	2.7	3.0	2.9	2.8	11.2	11.4	12.2	9.8	10.2	10.4	10.0	10.7	10.6	11.3	11.4	12.7	12.8	13.4	11.4	11.0	8.9	10.1	10.2	10.4	10.6	12.8	7.3	8.4
8. A/swine/Chile/VN1401-343/2014(H1N2)	3.8	3.1	3.9	3.2	3.2	3.2	0.5	0.0	0.0	3.4	3.0	3.4	3.2	3.2	11.2	11.4	12.2	9.4	9.8	10.0	9.6	10.3	10.2	10.9	11.0	12.3	12.3	13.0	11.0	10.7	8.4	9.7	9.8	10.0	10.2	12.4	6.8	7.8
9. A/swine/Chile/VN1401-367/2014(H1N2)	3.8	3.2	4.0	3.2	3.2	3.2	0.6	0.0	0.0	3.4	3.0	3.4	3.2	3.2	11.2	11.4	12.2	9.3	9.7	10.0	9.5	10.2	10.2	10.8	10.9	12.2	12.3	12.9	10.9	10.6	8.3	9.6	9.7	10.0	10.2	12.3	6.7	7.8
10. A/swine/Chile/389/2014(H1N2)	2.4	0.6	0.9	1.6	1.5	2.4	2.9	3.4	3.4	0.0	1.6	1.6	1.6	1.5	10.5	10.5	11.5	11.6	11.9	11.9	11.6	12.5	12.6	13.2	13.4	14.8	14.8	15.4	12.9	12.4	11.5	11.7	11.9	11.9	12.1	14.7	9.8	11.0
11. A/swine/Chile/454/2014(H1N2)	1.0	1.0	1.4	0.2	0.2	0.8	2.7	3.0	3.0	1.6	0.0	0.4	0.2	0.2	9.1	9.2	10.1	10.1	10.4	10.3	10.1	11.0	11.2	11.7	12.0	13.4	13.3	13.9	11.3	10.8	10.5	10.2	10.4	10.3	10.6	13.2	8.7	10.0
12. A/swine/Chile/482/2014(H1N2)	0.8	1.0	1.2	0.2	0.2	0.9	3.0	3.4	3.4	1.6	0.4	0.0	0.2	0.2	9.0	9.0	10.0	10.3	10.6	10.5	10.2	11.2	11.4	11.9	12.2	13.7	13.6	14.2	11.5	10.9	10.8	10.4	10.6	10.5	10.8	13.4	9.0	10.3
13. A/swine/Chile/694/2015(H1N2)	0.9	1.0	1.3	0.0	0.1	0.8	2.9	3.2	3.2	1.6	0.2	0.2	0.0	0.1	9.0	9.1	10.0	10.2	10.5	10.4	10.1	11.1	11.3	11.8	12.1	13.6	13.4	14.0	11.4	10.9	10.6	10.3	10.5	10.4	10.7	13.3	8.9	10.1
14. A/swine/Rancagua/VN1401-699/2015(H1N2)	0.9	0.9	1.2	0.1	0.0	0.9	2.8	3.2	3.2	1.5	0.2	0.2	0.1	0.0	9.1	9.2	10.1	10.3	10.5	10.5	10.2	11.2	11.3	11.8	12.2	13.6	13.5	14.1	11.5	10.9	10.7	10.3	10.5	10.5	10.7	13.3	8.9	10.2
15. A/swine/Mallarauco/VN1401-03/2014(H1N2)	8.2	10.0	9.7	9.0	9.1	8.4	11.2	11.2	11.2	10.5	9.1	9.0	9.0	9.1	0.0	0.5	1.0	9.8	9.5	8.7	9.0	10.1	10.9	10.7	11.6	13.0	12.3	12.6	9.2	8.3	13.8	9.1	9.5	8.7	8.9	11.7	12.2	13.5
16. A/swine/Chile/VN1401-4/2014(H1N2)	8.2	10.0	9.7	9.1	9.2	8.5	11.4	11.4	11.4	10.5	9.2	9.0	9.1	9.2	0.5	0.0	1.0	10.3	10.1	9.2	9.5	10.6	11.5	11.3	12.1	13.6	12.9	13.1	9.8	8.9	14.2	9.6	10.1	9.2	9.5	12.2	12.6	13.9
17. A/swine/Mallarauco/VN1401-988/2015(H1N2)	9.1	10.9	10.7	10.0	10.1	9.4	12.2	12.2	12.2	11.5	10.1	10.0	10.0	10.1	1.0	1.0	0.0	10.4	10.1	9.2	9.6	10.7	11.5	11.3	12.2	13.5	12.8	13.0	9.7	8.8	14.6	9.7	10.1	9.2	9.5	12.1	13.0	14.3
18. A/swine/Chile/VN1401-80/2014(H1N1)	9.8	11.0	11.5	10.2	10.3	9.4	9.8	9.4	9.3	11.6	10.1	10.3	10.2	10.3	9.8	10.3	10.4	0.0	0.5	1.5	0.9	0.9	1.3	1.6	2.1	3.6	3.3	3.8	2.0	2.3	5.1	0.9	0.5	1.5	1.4	3.1	4.4	5.0
19. A/swine/Chile/VN1401-82/2014(H1N1)	10.1	11.3	11.8	10.5	10.5	9.7	10.2	9.8	9.7	11.9	10.4	10.6	10.5	10.5	9.5	10.1	10.1	0.5	0.0	1.0	0.5	0.7	1.4	1.4	2.1	3.6	3.1	3.6	1.5	1.8	5.6	0.5	0.0	1.0	0.9	2.8	4.9	5.5
20. A/swine/Rengo/VN1401-95/2014(H1N1)	9.9	11.3	11.7	10.4	10.5	9.6	10.4	10.0	10.0	11.9	10.3	10.5	10.4	10.5	8.7	9.2	9.2	1.5	1.0	0.0	0.6	1.4	2.3	2.0	2.9	4.3	3.7	4.1	1.0	0.9	6.5	0.6	1.0	0.0	0.3	3.2	5.7	6.4
21. A/swine/Chile/VN1401-336/2014(H1N1)	9.7	11.0	11.4	10.1	10.2	9.3	10.0	9.6	9.5	11.6	10.1	10.2	10.1	10.2	9.0	9.5	9.6	0.9	0.5	0.6	0.0	1.2	2.0	1.9	2.6	4.1	3.6	4.1	1.4	1.5	5.9	0.1	0.5	0.6	0.7	3.2	5.1	5.8
22. A/swine/O'Higgins/VN1401-380/2014(H1N1)	10.7	11.9	12.4	11.1	11.2	10.3	10.7	10.3	10.2	12.5	11.0	11.2	11.1	11.2	10.1	10.6	10.7	0.9	0.7	1.4	1.2	0.0	0.9	0.7	1.5	3.0	2.5	3.0	1.5	2.1	5.5	1.1	0.7	1.4	1.3	2.2	5.0	5.5
23. A/swine/Chile/VN1401-508/2014(H1N1)	11.0	12.1	12.6	11.3	11.3	10.5	10.6	10.2	10.2	12.6	11.2	11.4	11.3	11.3	10.9	11.5	11.5	1.3	1.4	2.3	2.0	0.9	0.0	0.9	0.8	2.4	2.2	2.8	2.4	3.1	4.7	1.9	1.4	2.3	2.2	2.2	4.5	4.8
24. A/swine/Chile/VN1401-525/2014(H1N1)	11.4	12.6	13.1	11.8	11.8	11.0	11.3	10.9	10.8	13.2	11.7	11.9	11.8	11.8	10.7	11.3	11.3	1.6	1.4	2.0	1.9	0.7	0.9	0.0	0.9	2.3	1.7	2.3	1.7	2.6	5.6	1.8	1.4	2.0	1.8	1.5	5.3	5.7
25. A/swine/Chile/VN1401-536/2014(H1N1)	11.8	12.9	13.4	12.1	12.2	11.4	11.4	11.0	10.9	13.4	12.0	12.2	12.1	12.2	11.6	12.1	12.2	2.1	2.1	2.9	2.6	1.5	0.8	0.9	0.0	1.5	1.4	2.0	2.7	3.5	4.9	2.5	2.1	2.9	2.7	1.6	5.0	5.1
26. A/swine/Valparaiso/VN1401-559/2014(H1N1)	13.3	14.3	14.9	13.6	13.6	12.8	12.7	12.3	12.2	14.8	13.4	13.7	13.6	13.6	13.0	13.6	13.5	3.6	3.6	4.3	4.1	3.0	2.4	2.3	1.5	0.0	0.9	1.3	3.9	4.8	5.4	4.0	3.6	4.3	4.1	1.8	5.9	5.7
27. A/swine/Chile/VN1401-571/2014(H1N1)	13.1	14.2	14.7	13.4	13.5	12.7	12.8	12.3	12.3	14.8	13.3	13.6	13.4	13.5	12.3	12.9	12.8	3.3	3.1	3.7	3.6	2.5	2.2	1.7	1.4	0.9	0.0	0.7	3.1	4.0	6.0	3.5	3.1	3.7	3.5	0.9	6.3	6.2
28. A/swine/Chile/VN1401-587/2014(H1N1)	13.7	14.8	15.3	14.0	14.1	13.3	13.4	13.0	12.9	15.4	13.9	14.2	14.0	14.1	12.6	13.1	13.0	3.8	3.6	4.1	4.1	3.0	2.8	2.3	2.0	1.3	0.7	0.0	3.4	4.3	6.6	4.0	3.6	4.1	3.8	0.9	6.9	6.8
29. A/swine/Rancagua/VN1401-688/2015(H1N1)	10.9	12.3	12.7	11.4	11.5	10.6	11.4	11.0	10.9	12.9	11.3	11.5	11.4	11.5	9.2	9.8	9.7	2.0	1.5	1.0	1.4	1.5	2.4	1.7	2.7	3.9	3.1	3.4	0.0	0.9	6.9	1.4	1.5	1.0	0.8	2.5	6.4	7.0
30. A/swine/Rancagua/VN1401-689/2015(H1N1)	10.3	11.8	12.1	10.9	10.9	10.0	11.0	10.7	10.6	12.4	10.8	10.9	10.9	10.9	8.3	8.9	8.8	2.3	1.8	0.9	1.5	2.1	3.1	2.6	3.5	4.8	4.0	4.3	0.9	0.0	7.4	1.5	1.8	0.9	0.9	3.4	6.6	7.3
31. A/swine/Rancagua/VN1401-1107/2015(H1N1)	10.7	11.1	11.8	10.6	10.7	10.1	8.9	8.4	8.3	11.5	10.5	10.8	10.6	10.7	13.8	14.2	14.6	5.1	5.6	6.5	5.9	5.5	4.7	5.6	4.9	5.4	6.0	6.6	6.9	7.4	0.0	5.9	5.6	6.5	6.5	6.5	1.9	0.5
32. A/swine/Pelarco/VN1401-1308/2015(H1N1)	9.8	11.1	11.5	10.3	10.3	9.5	10.1	9.7	9.6	11.7	10.2	10.4	10.3	10.3	9.1	9.6	9.7	0.9	0.5	0.6	0.1	1.1	1.9	1.8	2.5	4.0	3.5	4.0	1.4	1.5	5.9	0.0	0.5	0.6	0.6	3.1	5.1	5.8
33. A/swine/Pelarco/VN1401-1309/2015(H1N1)	10.1	11.3	11.8	10.5	10.5	9.7	10.2	9.8	9.7	11.9	10.4	10.6	10.5	10.5	9.5	10.1	10.1	0.5	0.0	1.0	0.5	0.7	1.4	1.4	2.1	3.6	3.1	3.6	1.5	1.8	5.6	0.5	0.0	1.0	0.9	2.8	4.9	5.5
34. A/swine/Pelarco/VN1401-1314/2015(H1N1)	9.9	11.3	11.7	10.4	10.5	9.6	10.4	10.0	10.0	11.9	10.3	10.5	10.4	10.5	8.7	9.2	9.2	1.5	1.0	0.0	0.6	1.4	2.3	2.0	2.9	4.3	3.7	4.1	1.0	0.9	6.5	0.6	1.0	0.0	0.3	3.2	5.7	6.4
35. A/swine/Rengo/VN1401-1563/2015(H1N1)	10.2	11.5	11.9	10.7	10.7	9.9	10.6	10.2	10.2	12.1	10.6	10.8	10.7	10.7	8.9	9.5	9.5	1.4	0.9	0.3	0.7	1.3	2.2	1.8	2.7	4.1	3.5	3.8	0.8	0.9	6.5	0.6	0.9	0.3	0.0	2.9	5.7	6.4
36. A/swine/Rengo/VN1401-1568/2015(H1N1)	12.9	14.1	14.6	13.3	13.3	12.5	12.8	12.4	12.3	14.7	13.2	13.4	13.3	13.3	11.7	12.2	12.1	3.1	2.8	3.2	3.2	2.2	2.2	1.5	1.6	1.8	0.9	0.9	2.5	3.4	6.5	3.1	2.8	3.2	2.9	0.0	6.6	6.7
37. A/swine/Chillan/VN1401-1705/2015(H1N1)	8.8	9.4	10.0	8.9	8.9	8.3	7.3	6.8	6.7	9.8	8.7	9.0	8.9	8.9	12.2	12.6	13.0	4.4	4.9	5.7	5.1	5.0	4.5	5.3	5.0	5.9	6.3	6.9	6.4	6.6	1.9	5.1	4.9	5.7	5.7	6.6	0.0	1.4
38. A/swine/Chillan/VN1401-1719/2015(H1N1)	10.2	10.6	11.3	10.1	10.2	9.6	8.4	7.8	7.8	11.0	10.0	10.3	10.1	10.2	13.5	13.9	14.3	5.0	5.5	6.4	5.8	5.5	4.8	5.7	5.1	5.7	6.2	6.8	7.0	7.3	0.5	5.8	5.5	6.4	6.4	6.7	1.4	0.0
